# Bile Acid Binding Resins Improve Glucagon Receptor Agonist‐Mediated Weight Loss in Diet‐Induced Obese Mice

**DOI:** 10.1002/oby.70087

**Published:** 2025-11-14

**Authors:** Teayoun Kim, Mackenzie J. Pearson, Huixian Hong, Shelly Nason, Khadija Seck, Jessica Antipenko, Natalie Presedo, Richard DiMarchi, William C. Roell, Kirk Habegger

**Affiliations:** ^1^ Comprehensive Diabetes Center and Division of Endocrinology, Diabetes and Metabolism, Department of Medicine, University of Alabama at Birmingham Birmingham Alabama USA; ^2^ Eli Lilly and Company, Lilly Corporate Center Indianapolis Indiana USA; ^3^ Department of Chemistry Indiana University Bloomington Indiana USA

## Abstract

**Objective:**

Glucagon‐receptor (GCGR) agonism reguglates bile acid (BA) metabolism and promotes weight loss in diet‐induced obese (DIO) mice. Thus, we hypothesized that BA signaling contributes to GCGR‐stimulated weight loss.

**Methods:**

To test this hypothesis, we utilized BA‐binding resins (BARs; colesevelam [Colsv] and cholestyramine [Cstyr]) to prevent intestinal BA reuptake. DIO C57Bl/6J mice were administered the GCGR agonist IUB288 or vehicle, in the presence or absence of BARs.

**Results:**

To our surprise, combined IUB288 and Colsv treatment reduced body weight and food intake compared to IUB288 or Colsv treatment groups in high fat diet (HFD)‐fed mice. Moreover, acute IUB288 + Colsv treatment reduced fasting‐stimulated HFD, but not chow, intake compared to IUB288 or Colsv treatments alone. We observed improved glucose homeostasis and reduced plasma cholesterol with combined IUB288 and Colsv, but not Colsv alone. Excitingly, liver steatosis was suppressed with IUB288 but not Colsv alone, and this benefit was further enhanced with combined treatment. Plasma BA profiles were regulated by both IUB288 and Colsv with concomitant modulation of liver and ileum BA regulatory mRNA expression. Similar findings were observed with the first‐generation BAR Cstyr.

**Conclusions:**

Together, these studies suggest that BARs enhance the antiobesity effect of GCGR agonism in DIO mice, representing a novel antiobesity strategy.

## 1 Introduction

Obesity rates have tripled since the 1970s (World Health Organization), and its prevalence is expected to continue [1]. This chronic disease state increases the risks of comorbidities such as type 2 diabetes, cardiovascular disease, and multiple cancers [2]. Weight loss stimulated via diet, exercise, surgery, or pharmacological agents all increase quality of life [3, 4, 5] and protect against comorbid outcomes [6, 7]. Thus, considerable effort has been placed on identifying novel mechanisms that will be effective against obesity and obesity‐related comorbidities.

In addition to its canonical role as a counter‐regulatory factor in glucose homeostasis, glucagon is a crucial regulator of lipid metabolism and energy balance. These metabolic actions include suppression of food intake and stimulation of energy expenditure [8]. In agreement with this role, the addition of glucagon‐receptor (GCGR) agonism enhances the weight loss effect of other antiobesity agents (e.g., glucagon‐like peptide‐1 [GLP‐1]). Of note, chronic GCGR activation stimulates considerable weight loss and is associated with decreased mRNA expression of genes related to synthesis of bile acids (BA) and modified circulating BA species [9]. Mechanistically, we have observed that hepatic GCGR agonism increases energy expenditure, and its antiobesity effects are partially dependent on hepatic farnesoid X receptor (FXR). FXR is a ligand‐activated transcription factor that is activated by BA [10]. Thus, liver FXR acts as a sensor for BA and functions as a key regulator of BA homeostasis, lipid metabolism, inflammation, and glucose metabolism [11]. Consistent with these actions, our prior work uncovered that mice with hepatic *Fxr*‐deficiency (*Fxr* ^ΔLiver^ mice) are resistant to IUB288‐mediated increases in energy expenditure and weight loss [9].

BA are best known for their role as detergents facilitating the absorption of dietary fat and lipid‐soluble nutrients. BA are synthesized in the liver, secreted into the bile ducts, and enter the intestinal tract. More than 95% of BA are reabsorbed in the ileum and returned to the liver through enterohepatic circulation, whereas unabsorbed BA are excreted in the feces. Cholesterol is a necessary substrate for BA synthesis. Hence, BA binding resins (BARs; e.g., colesevelam [Colsv] and cholestyramine [Cstyr]) were developed to lower plasma cholesterol by reducing BA reabsorption and depleting cholesterol levels [12]. Preclinical studies suggest that BARs may prevent diet‐induced obesity (DIO) [13, 14], but their role in the reversal of existing obesity is less clear. Intriguingly, Colsv increases postprandial glucagon levels, suggesting cross talk between these systems and even a possible state of glucagon resistance [14]. Based on these reports, we hypothesized that BAR treatment would suppress the antiobesity effects of GCGR. Studies herein utilized BAR treatments to investigate the role of BA in these antiobesity effects of GCGR agonism.

## 2 Methods

### 2.1 Animal Models

All studies were approved by and performed according to the guidelines of the Institutional Animal Care and Use Committee of the University of Alabama at Birmingham (UAB). C57Bl/6J mice were obtained from Jackson Labs and housed on a 12:12‐h light–dark cycle (light on from 0600 to 1800 h) at 22°C and constant humidity. Mice were single housed for the acute studies described in Figure 2d–g and group‐housed (i.e., two to three mice/cage) for all other studies. Mice were fed a standard chow (Teklad LM‐485, 5.6% fat) or high‐fat diet (HFD, 58.0 kcal% fat; D12331, Research Diets) with free access to food and water, except as noted.

### 2.2 Chemicals

Colesevelam (Colsv) was a gift from Daiichi Sankyo Inc. Cholestyramine (Cstyr) was purchased from SANDOZ Novartis Co. Either Colsv or Cstyr was mixed with pulverized HFD (D12331) and pelleted. IUB288 is a long‐acting, potent, and selective GGCR agonist with a ~6.5 h half‐life [15, 16]. IUB288 was synthesized as previously described [15], diluted in sterile saline, and delivered via daily subcutaneous injections.

### 2.3 Plasma and Tissue Analyses

Tissue and plasma lipids were determined using Infinity Triglycerides (Thermo Scientific #TR22421) or Infinity Cholesterol (Thermo Scientific #TR13421) assays. Hepatic lipid measurements were conducted following extraction as previously described [17, 18]. Plasma insulin, GLP‐1, and leptin were measured via ELISA (all from Crystal Chem). Histopathology for hematoxylin and eosin (H&E) liver staining was conducted as previously described [19]. Briefly, tissue was fixed for 12 h in 4% paraformaldehyde diluted in PBS at 4°C, embedded in paraffin, and cut at 4 μm for H&E staining. Slides were imaged using an Olympus IX81 fluorescence microscope and processed using the count and measure feature on CellSens Dimension software version 4.1.1 (Olympus).

### 2.4 BA Profiling

Plasma aliquots (50 μL) were extracted using methanol to recover BA. BA analytes were resolved using a Phenomenex XB‐C18 1.7 μm 2.1 × 100 mm LC column and detected on a SCIEX 7500 system. BA peak areas were analyzed by SCIEX OS Analytics 3.1.6, and calculated concentrations were reported based on analyte area/internal standard area ratios based on a standard curve for each analyte.

### 2.5 Body Composition

Body weight and food intake measurements were collected twice a week. Body composition was measured using magnetic resonance spectroscopy (EchoMRI, Echo Medical Systems) in the Nutrition Obesity Research Center Small Animal Phenotyping Core at UAB.

### 2.6 Quantitative Real‐Time PCR

Tissue RNA was isolated using the RNeasy Lipid Mini Kit according to the manufacturer's instructions (Qiagen). cDNA was synthesized by reverse transcription PCR using SuperScript III, DNase treatment, and anti‐RNase treatment according to the manufacturer's instructions (Invitrogen). Single gene quantitative PCR was performed as previously described [17]. Data were normalized to housekeeping gene *Rps18* using the ΔΔct calculation. The primer sequences are listed below in Table 1.

**TABLE 1:** List of primer sets for quantitative real‐time PCR.

Primer names	Primer sequences
*Gcg* Forward	ATC TTG CCA CCA GGG ACT TC
*Gcg* Reverse	AAG TGA CTG GCA CGA GAT GT
*Ppargc1a* Forward	CCC TGC CAT TGT TAA GAC C
*Ppargc1a* Reverse	TGC TGC TGT TCC TGT TTT C
*Cyp7a1* Forward	GGG ATT GCT GTG GTA GTG AGC
*Cyp7a1* Reverse	GGT ATG GAA TCA ACC CGT TGT C
*Cyp8b1* Forward	CCA GTA CTT CAC CTT TGT CAT GGA C
*Cyp8b1* Reverse	GTA CCC AAA CAC CTT GAG CAC CAG TTC
*Cyp27a1* Forward	CGT TTA AGG CAT CCG TGT AGA GCG
*Cyp27a1* Reverse	GAG TAC ACA CCA GGG ACA CTG ATC CAG
*Shp* Forward	CAT GGC CTC TAC CCT CAA GAA C
*Shp* Reverse	GTC ACC TCA GCA AAA GCA TGT C
*Fgf15* Forward	GCC ATC AAG GAC GTC AGC A
*Fgf15* Reverse	CTT CCT CCG AGT AGC GAA TCA G
*Gpbar1* Forward	AAG AGC CAA GAG GGA CAA TC
*Gpbar1* Reverse	GTA GCT GCT GCT TCC CTA AT
*Slc2a1* Forward	GGG AGA CGC ATA GTT ACA GC
*Slc2a1* Reverse	CTC CCA CAG CCA ACA TGA G
*Rps18* Forward	TTC TGG CCA ACG GTC TAG ACA AC
*Rps18* Reverse	CCA GTG GTC TTG GTG TGC TGA

### 2.7 Statistics

All data are represented as mean and SEM. Statistical significance was determined using unpaired Student's *t‐*tests or, where appropriate, one‐ and two‐way ANOVA with multiple comparisons Tukey and Sidak posttests, respectively. Statistics were completed using GraphPad Prism version 10.5.0 for Macintosh and Windows (GraphPad Software). Statistical significance was assigned when *p* < 0.05.

## 3 Results

### 3.1 Colesevelam Enhances GCGR‐Mediated Body Weight Loss

**FIGURE 1:**
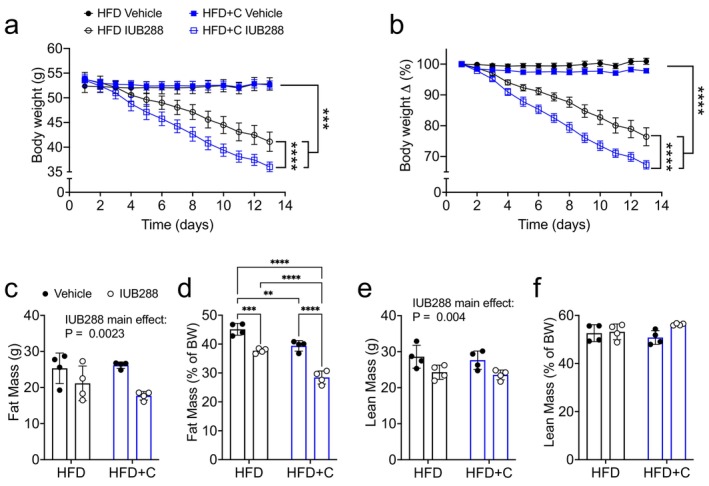
Colesevelam (2%) enhances GCGR‐mediated body weight loss in DIO mice. (a) Body weight, (b) body weight change (%), (c) fat mass, (d) %fat mass, (e) lean mass, and (f) %lean mass in DIO mice treated for 13 days with IUB288 (10 nmol/kg/d), Colsv (2% in HFD), or both in combination. All data are represented as mean ± SEM in 6‐month‐old C57Bl/6J mice (*n* = 11 for panels a and b and 4 panels for c–f). **p < 0.01, ***p < 0.001 and ****p < 0.0001 as analyzed by two‐way RM‐ANOVA (a, b) or standard two‐way ANOVA (c–e). [Color figure can be viewed at wileyonlinelibrary.com]

We utilized the BAR Colsv to interrogate BA as potential mediators of GCGR‐stimulated weight loss in DIO mice. To establish DIO, 10‐week‐old male C57Bl/6J mice were provided ad libitum access to HFD for 12 weeks. DIO mice were randomized into two groups matched for body weight. Mice were provided continuous access to either HFD or HFD supplemented with 2% Colsv (HFD + C), a dose shown to improve glucose homeostasis in DIO mice [14]. HFD + C was provided to mice for a 7‐day pretreatment period to allow for stabilization of any effects on energy balance or glucose homeostasis prior to IUB288 treatment. Mice from each group were then treated with either IUB288 (10 nmol/kg s.c.) or vehicle (isotonic saline) daily for 14 days. Vehicle‐treated mice maintained body weight when fed either HFD or HFD + C (Figure 1a,b), while daily IUB288 treatment stimulated weight loss over the 14‐day treatment (Figure 1a,b). Contrary to our hypothesis, IUB288 treatment in HFD + C‐fed mice stimulated greater weight loss than in IUB288‐treated HFD‐fed mice (Figure 1a,b).

Body composition was measured via quantitative magnetic resonance on Day 13 in a subset (*n* = 4/group) of these mice. IUB288 treatment reduced absolute and relative (%) fat mass as compared to HFD + Vehicle mice (Figure 1c,d). We also observed a decrease in % fat mass of HFD + C mice as compared to HFD + Vehicle controls (Figure 1d). However, the largest fat mass reduction was observed in the combined IUB288 + C treated mice, which were leaner than all other groups (Figure 1d). IUB288 also reduced absolute lean mass irrespective of Colsv over the treatment period (Figure 1e). However, this lean mass loss should be considered in the context of whole body weight loss. Thus, when normalized to total body weight, we uncovered similar levels of % lean mass across all treatments (Figure 1f).

### 3.2 Colesevelam Enhances GCGR‐Mediated Hypophagia

**FIGURE 2:**
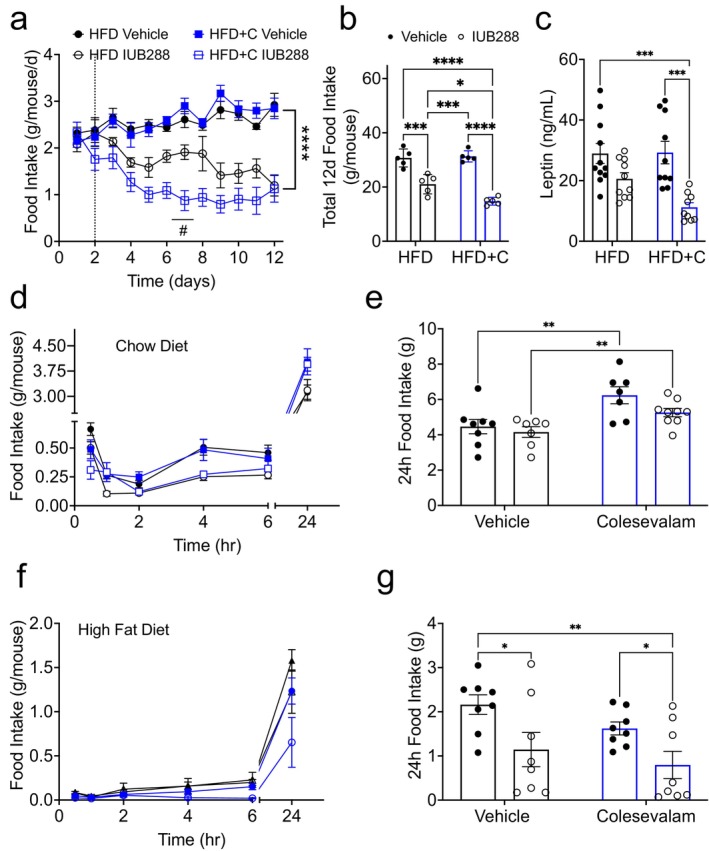
Colesevelam (2%) enhances GCGR‐mediated hypophagia in DIO mice. (a) Daily food intake (b) and total 12‐day food intake in DIO mice treated for 13 days with IUB288 (10 nmol/kg/d), Colsv (2% in HFD), or both in combination. (c) 2‐h fasted plasma leptin from Day 13. Fasting‐stimulated food intake in (d, e) chow‐ or (f, g) HFD‐fed mice. Colsv (10 mg p.o.) was delivered to 16‐h fasted mice immediately before the reintroduction of each diet. All data are represented as mean ± SEM (*n* = 8) in 4‐month‐old C57Bl/6J mice. Mice were provided HFD immediately following Colsv gavage. *p < 0.05, **p < 0.01, ***p < 0.001 and ****p < 0.0001 as analyzed by two‐way ANOVA. [Color figure can be viewed at wileyonlinelibrary.com]

Daily home‐cage food intake was measured in all mice. Consistent with our prior reports, we observed reduced food intake in IUB288‐treated mice (Figure 2a,b). Colsv alone had no effect on food intake in our mice; however, when combined with IUB288, Colsv provided additional suppression of food intake (Figure 2a,b). As expected with reduced fat mass, leptin levels were lower in IUB288‐treated groups compared to vehicle, with the greatest effect occurring in the IUB288 + Colsv group compared to HFD + Colsv and vehicle only groups (treatment effect *p* = 0.01; Figure 2c).

Our data suggest that a portion of the body weight lost in IUB288 + Colsv treatment is due to suppression of food intake. However, this hypophagia was observed in the context of chronic treatment. To assess the acute effect of BA sequestration on food intake, we delivered 10 mg of Colsv (in saline) via gavage to overnight (16 h)‐fasted mice maintained on chow or HFD. Importantly, this strategy also controls for potential changes in food intake that may be induced when Colsv is delivered in the diet. Colsv was administered immediately prior to refeeding, and food intake was measured in each mouse at 0.5, 1, 2, 4, 6, and 24 h. Surprisingly, 24‐h food intake in chow‐fed mice was unchanged by IUB288, while Colsv increased food intake regardless of IUB288 treatment (Figure 2d,e). To assess the acute effect of BA sequestration on HFD intake, we repeated this experimental paradigm but provided HFD at the time of refeeding. We observed that in HFD‐fed mice, food intake was unchanged by Colsv gavage (*p* = 0.54) but reduced by IUB288 treatment (Figure 2f,g). Moreover, Colsv and IUB288 co‐treatment in HFD‐fed mice uncovered reduced food intake as compared to Colsv alone (Figure 2f,g). Together, these data suggest that combined IUB288 and Colsv treatment decreases food intake in HFD‐, but not chow‐fed, mice.

### 3.3 Colesevelam Enhances GCGR‐Mediated Regulation of Glucose and Lipid Homeostasis

**FIGURE 3:**
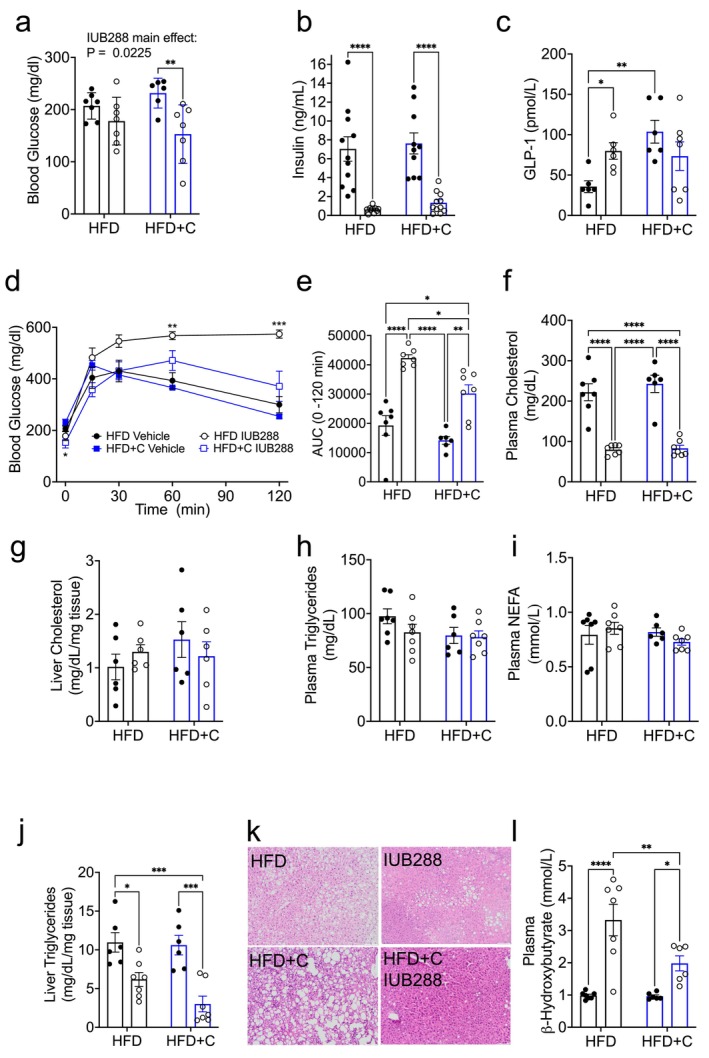
Colesevelam (2%) and GCGR agonism regulate glucose and lipid homeostasis in DIO mice. (a) 6‐h fasting blood glucose and (b) insulin prior to ipGTT on Day 12. (c) Plasma glucagon‐like peptide‐1 (GLP‐1) in 2‐h fasted mice on Day 13. (d, e) Glucose excursion and area under the curve (AUC) analysis from ipGTT. (f, g) Plasma and liver cholesterol and (h, i) plasma triglycerides and nonesterified fatty acids (NEFA) from study termination (Day 13). (j, k) Liver triglycerides and representative H&E staining from study termination (scale bar = 100 μm). (l) Plasma β‐hydroxybutyratefrom study termination. All data are represented as mean ± SEM in 6‐month‐old C57Bl/6J mice from Figure 1 (*n* = 6–7). *p < 0.05, **p < 0.01, ***p < 0.001, and ****p < 0.0001 as analyzed by standard two‐way ANOVA (a–c and e–l) or two‐way RM‐ANOVA (d). [Color figure can be viewed at wileyonlinelibrary.com]

We next assessed glucose and lipid homeostasis following chronic (13 days) Colsv and IUB288 treatment in DIO mice (Figure 3a–l). Fasting (5 h) blood glucose and plasma insulin were reduced in all IUB288‐treated groups (main effect *p* = 0.0225; Figure 3a,b). This reduction in insulin was of interest as both IUB288 and Colsv independently increased circulating GLP‐1 in 2‐h fasted mice (Figure 3c). When these mice were administered an i.p. glucose challenge (1.5 g/kg body weight, 24 h after the last injection), we observed impaired glucose tolerance in the IUB288‐treated mice (Figure 3d,e). However, this IUB288‐induced glucose intolerance was considerably improved in the presence of Colsv (Figure 3d,e). Plasma cholesterol assessed at study termination was greatly reduced in IUB288‐treated mice (Figure 3f), an effect consistent with our prior reports [9, 15, 17, 18]. While plasma cholesterol in the HFD + C mice was similar to HFD control mice, robust suppression was observed in IUB288 alone and IUB288 + Colsv treatment groups (Figure 3f). Conversely, liver cholesterol levels were not altered by these treatments (Figure 3g). Likewise, circulating levels of nonesterified fatty acids (NEFA) and triglycerides were similar in all treatment groups (Figure 3h,i). As we have previously reported, GCGR agonism is profoundly antisteatotic [9, 15, 17, 18]. Consistent with those findings, liver triglyceride levels were reduced by IUB288 treatment as compared to HFD control mice (Figure 3j). Although liver triglyceride levels in the HFD + C mice were similar to HFD control mice, we observed considerable reduction in IUB288 + Colsv mice. Moreover, there was added benefit in combined IUB288 + Colsv treatment as compared to IUB288 alone (Figure 3j; *p* = 0.0367, *t*‐test). H&E staining of these livers confirmed reduced liver steatosis following IUB288 treatment that was enhanced when combined with Colsv (Figure 3k). Analysis of plasma β‐hydroxybutyrate uncovered a significant profound increase in this ketone, and marker of liver fatty acid oxidation, in IUB288‐treated mice. However, combined IUB288 + Colsv treatment significantly reduced β‐hydroxybutyrate levels compared to IUB288 treatment alone (Figure 3l). Together these data support a reversal of DIO‐associated liver steatosis and hypercholesterolemia following GCGR agonism.

### 3.4 Colesevelam and GCGR‐Mediated Regulation of BA Profile and BA‐Regulatory Genes in Ilium and Liver

**FIGURE 4:**
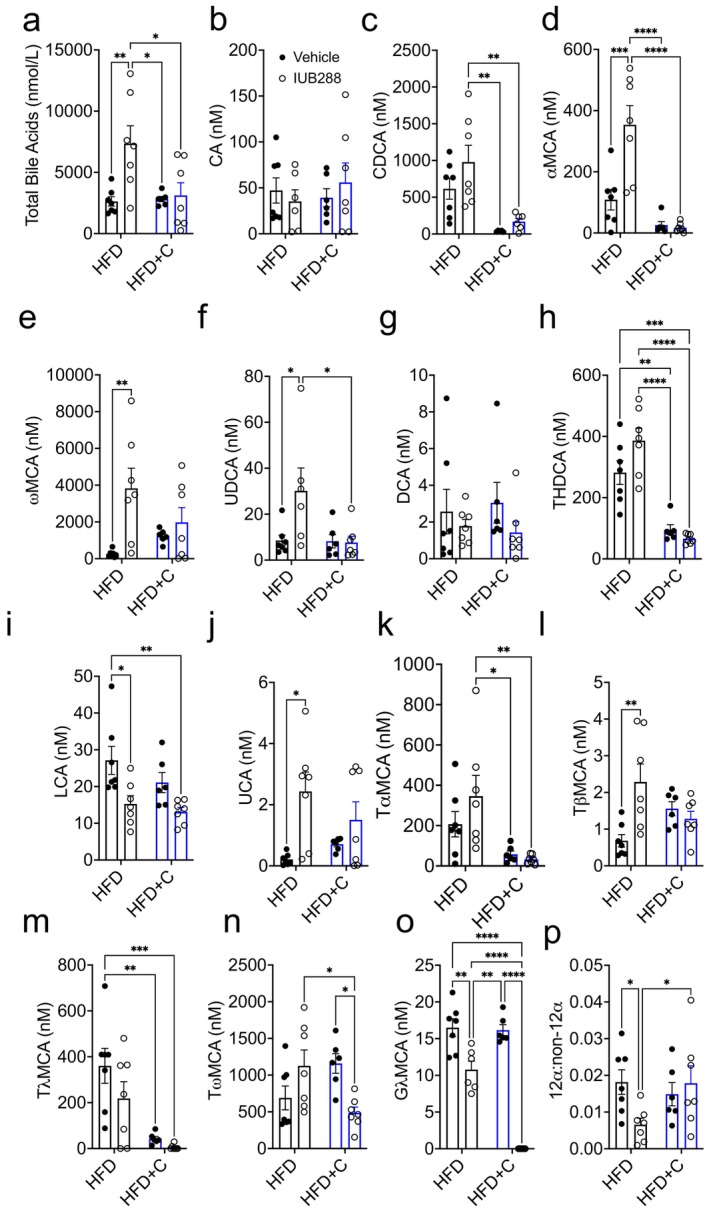
Colesevelam (2%) and GCGR‐mediated bile acid profiles in DIO mice. (a) Total bile acids, (b) cholic acid (CA), (c) chenodeoxycholic acid (CDCA), (d) α‐muricholic acid (α‐MCA), (e) ω‐muricholic acid (ω‐MCA), (f) ursodeoxycholic acid (UDCA), (g) deoxycholic acid (DCA), (h) taurohyodeoxycholic acid (THDCA), (i) lithocholic acid (LCA), (j) ursocholic acid (UCA), (k) tauro‐α‐MCA (T‐α‐MCA), (l) T‐β‐MCA, (m) T‐λ‐MCA, (n) T‐ω‐MCA, and (o) gly‐λ‐MCA from plasma collected at study termination. (p) Ratio of 12α‐hydroxylated to non‐12α‐hydroxylated bile acids. All data are represented as mean ± SEM in 6‐month‐old C57Bl/6J mice from Figure 1 (*n* = 6–7). *p < 0.05, **p < 0.01, ***p < 0.001, and ****p < 0.0001 as analyzed by standard two‐way ANOVA. [Color figure can be viewed at wileyonlinelibrary.com]

GCGR‐signaling is a crucial regulator of BA metabolism [8]. Consistent with our prior reports [9], chronic GCGR agonism via IUB288 increases total circulating levels of BA (Figure 4a). Although Colsv treatment alone had no effect on circulating BA levels, this treatment was sufficient to prevent the IUB288‐induced increase in plasma BA (Figure 4a). Regarding changes in individual BA, Colsv treatment reduced plasma levels of chenodeoxycholic acid (CDCA), α‐muricholic acid (α‐MCA), taurohyodeoxycholic acid (THDCA), tauro‐α‐MCA, and tauro‐λ‐MCA (Figure 3c,d,h,k,m). Intriguingly, Colsv treatment had no effect on plasma cholic acid (CA), ω‐muricholic acid (ω‐MCA), ursodeoxycholic acid (UDCA), deoxycholic acid (DCA), lithocholic acid (LCA), ursocholic acid (UCA), tauro‐β‐MCA, tauro‐ω‐MCA, or gly‐λ‐MCA (Figure 4b,e–g,i,j,l,n,o), suggesting these BA species are resistant to Colsv binding. IUB288 treatment had no effect on circulating CA, CDCA, DCA, THDCA, tauro‐α‐MCA, tauro‐λ‐MCA, or tauro‐ω‐MCA levels (Figure 4b,c,g,h,k,m,n). Converse to Colsv, IUB288 treatment increased circulating levels of α‐MCA, ω‐MCA, UDCA, UCA, and tauro‐β‐MCA (Figure 4d–f,j,l). IUB288 reduced lithocholic acid (LCA), but the combination had no additional effect (Figure 4i). However, we did uncover synergistic suppression of tauro‐λ‐MCA and gly‐λ‐MCA (Figure 4m–o). Finally, when calculating the ratio of circulating 12α‐hydroxylated to non‐12α‐hydroxylated BA, we observed an interaction between the main effects for Colsv and IUB288 treatment (*p* = 0.0450; Figure 4p).

**FIGURE 5:**
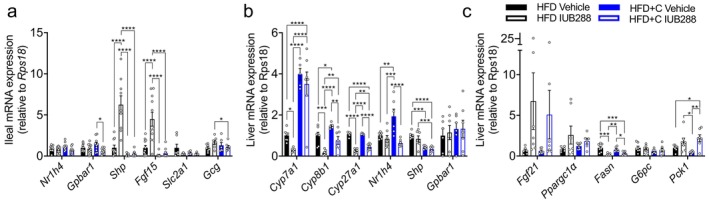
Transcription of ileal and liver bile acid regulatory mRNA by colesevelam (2%) and GCGR agonism in DIO mice. (a) Ileal and (b, c) liver mRNA expression of bile acid and lipo‐regulatory genes in tissues collected at study termination. All data are represented as mean ± SEM in 6‐month‐old C57Bl/6J mice from Figure 1 (*n* = 7–11). *p < 0.05, **p < 0.01, ***p < 0.001, and ****p < 0.0001 as analyzed by standard two‐way ANOVA. [Color figure can be viewed at wileyonlinelibrary.com]

We also measured mRNA expression of BA‐regulatory genes in the ilium (Figure 5a) and liver (Figure 5b,c). Ileal expression of BA receptors farnesoid X receptor (*Nr1h4*) and G‐protein‐coupled BA receptor 1 (*Gpbar1*) was not altered by IUB288 treatment (Figure 5a). However, IUB288 considerably elevated expression of small heterodimer partner (*Shp or Nr0b2*) and fibroblast growth factor 15 (*Fgf15*; Figure 5a). This expression pattern is consistent with increased ileal BA exposure [20] and our prior description of IUB288‐stimulated FXR effects [9]. As expected with BA sequestration, we observed complete suppression of the IUB288 induction at *Shp* and *Fgf15* in Colsv‐treated mice (Figure 5a). While we (Figure 3c) and others [14] have observed increased GLP‐1 following Colsv treatment, ileal proglucagon (*Gcg*) mRNA was not regulated in our mice, nor did we observe changes in glucose transporter 1 (Slc2a1; Figure 5a).

Assessment of liver mRNA expression uncovered robust regulation of genes associated with BA synthesis. Specifically, IUB288 treatment suppressed mRNA expression of cytochrome P450 family 7 subfamily A member 1 (*Cyp7a1*), *Cyp8b1*, and *Cyp27a1* (Figure 5b). However, combined IUB288 + Colsv treatment reversed these effects (Figure 5b). Thus, Colsv treatment was sufficient to overcome the transcriptional suppression of these genes induced by GCGR agonism (Figure 5b). Liver expression of BA receptors *Nr1h4* (i.e., *Fxr*) and *Gpbar1* was not altered by IUB288 treatment (Figure 5b). Colsv treatment increased *Nr1h4* expression, which was blocked by IUB288 co‐treatment. Colsv treatment suppressed *Shp* expression, with similar effects observed following IUB288 co‐treatment (Figure 5b). We observed IUB288‐associated increases in *Fgf21* and *Pck1* (IUB288 main effect *p* = 0.04 and *p* = 0.0011, respectively), with similar trends in *Ppargc1α* (Figure 5c; IUB288 main effect *p* = 0.0909). Conversely, fatty acid synthase (*Fasn*) expression was suppressed by IUB288 (Figure 5c). Together, these data suggest a state of increased liver fatty acid oxidation and reduced liver lipogenesis, providing insight into the reduced liver steatosis observed in our mice.

### 3.5 Cholestyramine Improves Circulating Lipid Profile by Increasing Hepatic Lipid Metabolism

**FIGURE 6:**
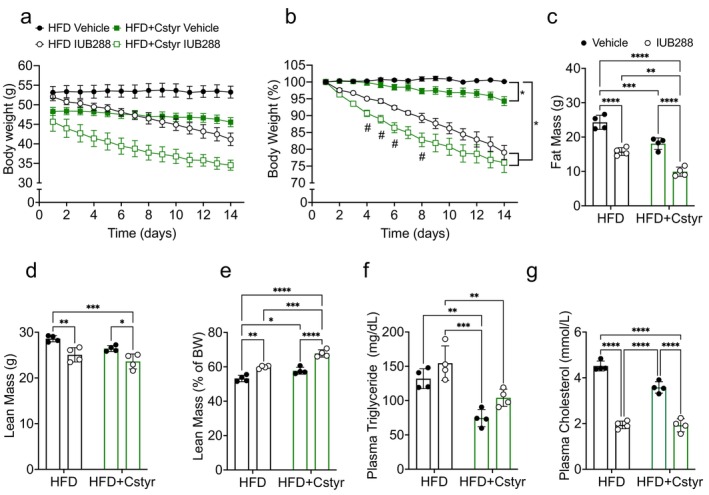
Cholestyramine (3%) enhances GCGR‐mediated body weight loss in DIO mice. (a) Body weight, (b) body weight change (%), (c) fat mass, (d) lean mass, and (e) lean mass change (% of body weight) in DIO mice treated for 14 days with IUB288 (10 nmol/kg/d), Cstyr (3% in HFD), or both in combination. Plasma (f) triglycerides and (g) cholesterol from *ad libitum* mice at study termination (Day 14). All data are represented as mean ± SEM (*n* = 4) in 3‐month‐old C57Bl/6J mice. *p < 0.05, **p < 0.01, ***p < 0.001, and ****p < 0.0001 as analyzed by two‐way RM‐ANOVA (a, b) or standard two‐way ANOVA (c–g). [Color figure can be viewed at wileyonlinelibrary.com]

To exclude possible off‐target effects of Colsv in the enhanced IUB288‐stimulated weight loss, we also tested another BAR, Cstyr. As with Colsv, Cstyr (3%) was delivered to DIO mice in HFD, and IUB288 (10 nmol/kg) or vehicle was administered daily via i.p. injection. IUB288 treatment for 14 days stimulated weight loss in DIO mice, with a smaller effect for weight loss observed in Cstyr as compared to vehicle controls (Figure 6a,b). As with Colsv, the combination of Cstyr and IUB288 stimulated considerable weight loss. This weight loss was greater than IUB288 alone, especially early in the treatment (Figure 6a,b, Days 4–8). These body weight changes were associated with reduced fat and lean mass (Figure 6c,d). However, as in the Colsv study, the greater proportion of weight loss was attributed to fat mass, resulting in an increase in the leanness (i.e., % lean mass) of these mice (Figure 6e).

Circulating triglyceride and cholesterol levels were assessed at study termination (Day 14) in *ad libitum* mice. Plasma triglyceride levels were unchanged by IUB288 treatment and were decreased by Cstyr (Figure 6f). However, the benefits stimulated by Cstyr alone were not enhanced by the combined IUB288–Cstyr treatment (Figure 6f). As expected from a BA sequestrant, we observed decreased plasma cholesterol in Cstyr‐fed mice (Figure 6g). IUB288 likewise stimulated a considerable reduction in plasma cholesterol that was not further reduced by the combined treatment (Figure 6g).

## 4 Discussion

Previously we reported that glucagon increases energy expenditure and that a portion of this effect is mediated via the BA nuclear receptor FXR and its signaling pathway in the liver [9]. Based on these observations, we attempted to remove this signal utilizing BARs, hypothesizing that this would subsequently reduce IUB288‐mediated weight loss. Contrary to this hypothesis, data herein detail enhanced weight loss in DIO mice following combined IUB288 and BAR treatment. We also observed an unexpected prevention of GCGR‐induced glucose intolerance as well as enhanced rescue from diet‐induced steatosis in these mice. Together these data provide exciting and enhanced therapeutic potential for GCGR agonism in metabolic diseases.

Orally administrated BARs are nonabsorbable, positively charged molecules (at intestinal pH). BARs bind the negatively charged BA within the intestinal lumen to inhibit reuptake, thus lowering BA pools while increasing fecal excretion [21]. The first‐generation BARs Cstyr and colestipol work exclusively in a charge‐specific manner, whereas the second‐generation BARs colestimide and Colsv employ both charge and hydrophobicity in their BA binding [22]. This strategy gives the second‐generation BARs five times greater affinity for BA over their predecessors [22]. Importantly, in these studies we utilized BARs from both generations (i.e., Cstyr and Colsv) and observed similar IUB288‐sensitizing effects on weight loss. Our interrogation of BA profiles uncovered a general increase in circulating BA in response to IUB288, with a reciprocal suppression when mice were treated with Colsv. The BA receptors FXR and TGR5 are both activated by lithocholic acid (LCA), deoxycholic acid (DCA), chenodeoxycholic acid (CDCA), and cholic acid (CA) [11, 23], while taurolithocholic acid (TLCA) and ursodeoxycholic acid (UDCA) are more specific ligands of TGR5 [23, 24]. Our analyzes did not identify a specific BA that might be preferentially activating these receptors in the combined IUB288 and Colsv treatments. Thus, the enhanced weight loss effects may be attributed to their reciprocal regulation, stimulating a futile cycle of IUB288‐stimulated synthesis and Colsv‐mediated disposal. The ratio of circulating 12α‐hydroxylated to non‐12α‐hydroxylated BA has been proposed as a marker of liver steatosis [25]. IUB288 treatment reduced this ratio, reinforcing this association. However, combined treatments blocked the IUB288 effect in spite of reduced liver triglycerides and histological steatosis in the combined group. Thus, our data suggest that this biomarker may be less useful in treatments that directly modulate BA homeostasis.

Improved metabolic control following BAR administration has been previously described. Of note, Colsv reduced hepatic glucose production via TGR5 and GLP‐1 in obese mice [14]. However, in our study, we observed increased plasma GLP‐1 in IUB288‐ and Colsv‐treated mice. However, these levels were not further elevated in the combined treatment group, and thus, GLP‐1 is unlikely to account for the enhanced weight loss effect. Regarding energy balance, colestimide stimulates energy expenditure in mice via increased cholesterol and BA synthesis in the liver and thermogenesis in brown adipose tissue [26]. Colsv likewise regulates various pools of BA. Unsurprisingly, fecal BA output is dramatically increased following Colsv treatment [27]. Consistent with its prevention of reabsorption, Colsv likewise reduces plasma BA in both lean and db/db mice [27]. However, BA synthesis is considerably increased, and thus, the total bile salt pool size is unchanged by Colsv [27]. Moreover, these data support that the divergent effects on BA homeostasis may contribute to the enhanced weight loss observed in our studies. Our studies were not designed as a direct comparison of the two BARs (i.e., Colsv and Cstyr). However, when combined with chronic GCGR agonism, both BARs reduced body weight in obese mice to a similar extent.

MASLD affects over 1 billion people worldwide, yet there is only a single approved treatment (i.e., resmetirom) available that directly targets this pathology [28]. MASLD can also progress to the more severe MASH, characterized by inflammation, hepatocyte injury, and fibrosis, increasing the risk for cirrhosis, hepatocellular carcinoma, and death [29]. Recent clinical evaluation of therapies that engage GCGR (e.g., retatrutide, cotadutide, survodutide, efinopegdutide) provides compelling evidence for the use of GCGR agonism against MASLD/MASH [30, 31, 32, 33]. Conversely, genetic (inactivating) variants in GCGR are associated with severe hepatic steatosis that was resolved upon liver transplant [34]. While it is yet unclear how targeting BA addresses MALSD/MASH, BARs have been utilized in the treatment of MASLD [35, 36]. Our current findings support the use of these two divergent therapies in combined treatment for metabolic liver disease.

There are several limitations to the current study. First, the systemic blood BA composition is significantly different from that in the enterohepatic circulation [37], which was not assessed in these mice. Future studies will interrogate the various BA pools in the context of GCGR agonism and BARs. Additionally, we did not directly assess energy expenditure in this study. However, the robust hypophagic effects observed following combined IUB288 and BAR treatments suggest that reduced energy intake rather than increased energy expenditure is the most likely driver of energy balance in these mice. Future studies will utilize indirect calorimetry and pair‐fed controls to further interrogate energy balance in these mice. Finally, these studies were restricted to male mice, and future studies will be required to assess the effect in females.

These current findings add to the growing appreciation of GCGR‐dependent physiology. These results also provide a deeper understanding of the GCGR‐based benefits in energy balance observed in coagonists [3, 4, 5, 35]. In sum, these data suggest that GCGR agonism reduces body weight via modulation of FXR‐dependent BA homeostasis. Moreover, sequestration of these BA can potentiate this GCGR‐dependent weight loss.

## Author Contributions

T.K., S.N., and K.H. were responsible for study conception and design, data analyses and interpretation, and drafting the article. H.H., M.J.P., T.K., S.N., N.P., K.S., and J.A. generated experimental data. R.D. and W.C.R. advised study concept and critical revision of the article. K.H. is the guarantor of this work and, as such, had full access to all the data in the study and takes responsibility for the integrity of the data and the accuracy of the data analysis.

## Conflicts of Interest

M.J.P. and W.C.R. are employees and shareholders of Eli Lilly and Company. The other authors declare no conflicts of interest.

## Data Availability

The data that support the findings of this study are available on request from the corresponding author. The data are not publicly available due to privacy or ethical restrictions.
